# Venoarteriovenous ECMO in Concomitant Acute Respiratory Distress Syndrome and Cardiomyopathy Associated with COVID-19 Infection

**DOI:** 10.1155/2021/8848013

**Published:** 2021-01-25

**Authors:** Zachary R. Bergman, Saranya Prathibha, Brent D. Bauman, Demetris Yannopoulos, Melissa E. Brunsvold

**Affiliations:** ^1^Department of Surgery, University of Minnesota, Minneapolis, MN, USA; ^2^Department of Surgery, Division of Acute Care Surgery and Critical Care, University of Minnesota, Minneapolis, MN, USA; ^3^Department of Internal Medicine, University of Minnesota, Minneapolis, MN, USA

## Abstract

In the most severe cases, novel coronavirus (SARS-CoV-2) infection leads to Acute Respiratory Distress Syndrome which may be refractory to standard medical interventions including mechanical ventilation. There are growing reports of the use of venovenous (VV) extracorporeal membrane oxygenation (ECMO) in these cases. A subset of critically ill COVID-19 patients develops cardiomyopathy as well, manifested by cardiogenic shock with reduced ejection fraction, dysrhythmias, and subsequent increase in mortality. One strategy for managing ARDS with an element of cardiogenic shock is venoarteriovenous (VAV) ECMO. Less than 1% of the cases in the worldwide ELSO COVID-19 database employed any form of hybrid cannulation. To date, there has only been one reported case of patient salvage with arterial or partial arterial support. We present a case that demonstrates the potential role of VAV ECMO in the case of concomitant severe ARDS with cardiomyopathy in the setting of COVID-19 infection.

## 1. Introduction

The global pandemic caused by the novel coronavirus (SARS-CoV-2) has altered the current healthcare landscape dramatically. In the most severe cases, COVID-19 infection leads to Acute Respiratory Distress Syndrome, which may be refractory to standard medical interventions including mechanical ventilation. There are growing reports of the use of venovenous (VV) extracorporeal membrane oxygenation (ECMO) in these cases [1–3]. A subset of critically ill COVID-19 patients develops cardiomyopathy as well, manifested by cardiogenic shock with reduced ejection fraction, dysrhythmias, and subsequent increase in mortality [4, 5]. To date, there is no consensus for management of severe ARDS and concomitant cardiomyopathy. One strategy for managing ARDS with an element of cardiogenic shock is venoarteriovenous (VAV) ECMO [6, 7]. Less than 1% of the cases in the worldwide ELSO COVID-19 database employed any form of hybrid cannulation. None of the COVID-19 patients in a recent paper who received arterial or partial arterial support survived [8]. We present a case that demonstrates the role of VAV ECMO in severe ARDS with cardiomyopathy in COVID-19 infection.

## 2. Case Report

**Figure 1:**
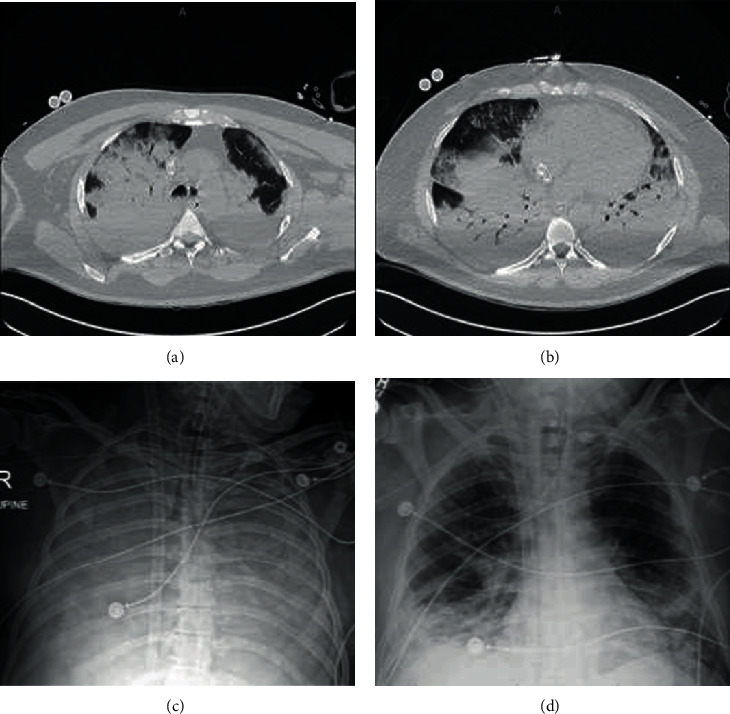
(a, b) Computed tomography of the chest immediately following ECMO cannulation (HD1) demonstrating dense consolidative opacities bilaterally. (c) Chest X-ray from HD8 demonstrating complete opacification of lungs bilaterally. (d) Chest X-ray from HD12 just prior to ECMO decannulation showing marked improvement aeration of bilateral lungs.

The patient was a nonsmoking 53-year-old male with a history of obesity (body mass index 32 kg/m^2^), type 2 diabetes mellitus, and hypertension who developed fevers, diarrhea, and cough. After 6 days of worsening symptoms, he presented to an outside hospital for rapidly worsening dyspnea and confusion. Upon arrival, he was found to have profound hypoxemia with an oxygen saturation of 33% by pulse oximetry. He was emergently intubated and admitted to the intensive care unit (ICU). Initial chest X-ray demonstrated mild bilateral pulmonary infiltrates (Figure 1). He was started on broad-spectrum antibiotics including vancomycin, piperacillin-tazobactam, and doxycycline. While in the ICU, his condition continued to worsen and he remained profoundly hypoxemic despite escalating ventilator support. Within five hours of hospital admission, he was on maximal ventilator settings, in the prone position, chemically paralyzed, and on inhaled epoprostenol. Despite these interventions, the patient remained severely hypoxic with oxygen saturation of 70% and PaO_2_ of 48 mmHg. Our institution was contacted to evaluate for initiation of VV ECMO. The patient was transferred to our surgical ICU still in a prone position and paralyzed.

On arrival to our surgical ICU, approximately 9 hours after his initial hospital presentation, his oxygen saturations were found to be between 40 and 60%, and he was increasingly hypotensive requiring norepinephrine and vasopressin to maintain an adequate blood pressure. His arterial blood gas identified a respiratory acidosis with a pH of 7.29 and elevated pCO_2_ to 59 mmHg. His PaO_2_ : FiO_2_ ratio was 36, consistent with severe ARDS. Initial management in our ICU included increasing PEEP to 24 cm while continuing 100% FiO_2_. Epinephrine was added as a third vasopressor for worsening hypotension. Despite these interventions, the PaO_2_ remained low at 44 mmHg and his oxygen saturation remained <70%. A bedside echocardiogram was obtained and identified globally diminished cardiac function with an estimated ejection fraction (EF) of 30%. His COVID-19 laboratory test returned positive soon after arrival to our institution.

**Figure 2:**
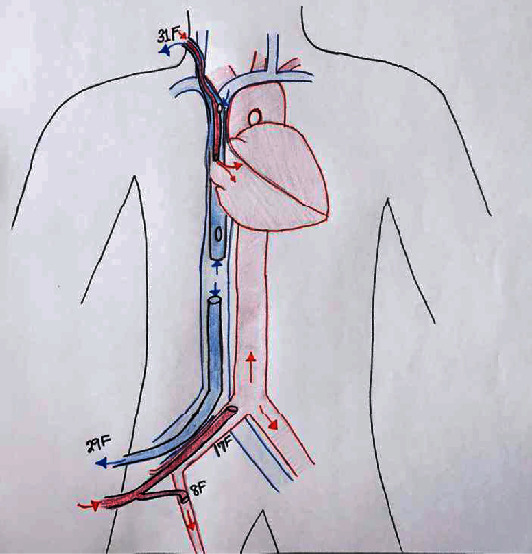
Complete VAV ECMO circuit.

Given the patient's ongoing profound hypoxemia and myocardial dysfunction with severely reduced EF, in collaboration with interventional cardiology, the decision was made to initiate venoarteriovenous (VAV) ECMO to provide oxygenation along with cardiac support for his stress cardiomyopathy. The patient was transferred to the interventional cardiology suite where coronary angiogram had no evidence of disease. A 17-French cannula was placed in the right femoral artery for arterial perfusion, a 29-French drainage cannula was placed in the right femoral vein, and an anterograde distal arterial perfusion 8-French catheter was inserted in the right superficial femoral artery to complete the VA circuit. A 31-French Avalon™ dual lumen venovenous cannula was then inserted in the right internal jugular vein, and the circuit was split with two “Y” connections to connect in parallel the VA and VV systems (Figure 2). Oxygen saturation immediately rose to 100%, and rest ventilator settings were implemented to prevent ventilator-induced lung injury.

After initiating ECMO therapy, his clinical condition gradually improved. His epoprostenol and chemically induced paralysis were discontinued, and all three vasopressors were quickly weaned off by HD 2 (ECMO day 1). His elevated troponin normalized after peaking at 1.953 mg/dL. A turndown study was completed and identified cardiac recovery with an EF of 40-45%. This was repeated on HD 3, and a normal EF was noted. During the turndown study, the femoral venous and arterial cannulas were clamped and the oxygen saturation was monitored. His oxygen saturation dropped from 100% to 82%, consistent with the decrease in total ECMO circuit flow from 5.5 liters per minute (LPM) to 4.6 LPM. With sustained hemodynamic stability and sustained oxygen saturation > 80% on VV ECMO (through the remaining right internal jugular dual lumen catheter), his femoral arterial and venous cannulas were removed through an open cut-down approach and vascular repair at the patient's bedside in the ICU (ECMO day 2). A heparin infusion was utilized for therapeutic anticoagulation while on ECMO targeting an activated clotting time of 160-180 seconds. While on VV ECMO, he was treated with piperacillin-tazobactam for empiric broad spectrum antibiotics for seven days.

**Figure 3:**
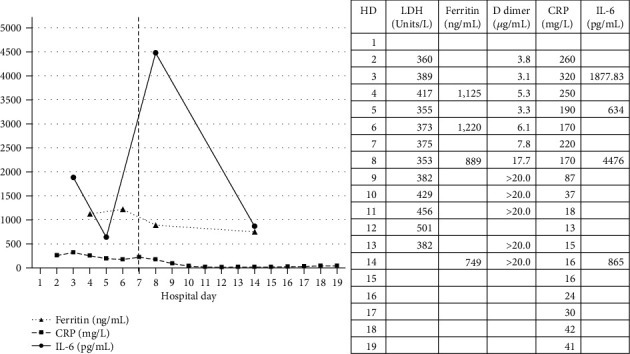
Inflammatory markers before and after sarilumab (IL-6 receptor inhibitor) on HD7 as shown by the vertical dotted line.

On HD 7, the patient was treated with a single dose of sarilumab 400 mg (IL-6 inhibitor) for ongoing increase in inflammatory cytokines with fever concerning for cytokine release syndrome with subsequent improvement in inflammatory markers (Figure 3.). Also on HD 7, he was noted to have worsening hypoxemia with oxygen saturation < 80% in a setting of high cardiac output of approximately 10 LPM. A transthoracic echocardiogram was also performed which revealed that the ECMO cannula had advanced to the IVC-RA junction with no flow towards the tricuspid valve. The cannula was therefore repositioned under echocardiographic guidance, and oxygen saturation slowly improved. He had dense consolidative opacities bilaterally on chest CT on the day of admission and worsening bilateral consolidative infiltrates on chest X-ray until HD 8, at which point, his oxygenation and chest imaging began to show improvement. His oxygenation gradually improved, and his ECMO sweep and flow rate was weaned until HD 12 (ECMO day 11) when he was decannulated after a 24-hour trial off sweep without ECMO support. After ECMO decannulation, he was maintained on rest ventilator settings until he was able to wean off the ventilator and was extubated on HD 14. He was able to gradually wean from the high-flow nasal cannula to room air. His COVID-19 RNA PCR test was repeated twice. After the return of a second negative COVID-19 RNA PCR test, airborne precautions were lifted, and he was able to ambulate in the hospital and was determined to be fit to discharge without the need for oxygen or support other than home physical therapy.

## 3. Discussion

The management of COVID-19-infected patients has been a perpetual learning process as the virus seems to affect nearly all the body's organ systems. In the critically ill, a multidisciplinary approach has been crucial to improve survival as pulmonary and renal failures are hallmarks of the disease and predictors of mortality [5]. Increasing evidence suggests that cardiomyopathy associated with the infection also influences mortality [3, 4]. For the majority of the cases, cardiomyopathy has been reported as a late complication of the infection. Our patient presented with concomitant respiratory failure and cardiomyopathy. It is possible that our patient's diminished cardiac function was driven by profound hypoxia as opposed to the proposed mechanism of cytokine release syndrome (CRS); however, by utilizing VAV ECMO, we were able to support him through his simultaneous pulmonary and cardiovascular collapse.

The combination of VA and VV ECMO circuits in this patient proved to be successful for a number of reasons. It is possible that with an EF of 30%, he would not have reached sufficient end-organ oxygenation with VV ECMO alone. The additional VA circuit was therefore able to provide cardiac support and improve end-organ oxygenation while awaiting myocardial recovery. Another advantage of VAV ECMO in our patient was the ability to prevent north-south syndrome from increasing antegrade cardiac output from the rapidly recovering heart opposing the retrograde oxygenated blood from the VA ECMO circuit [9]. By adding the VV circuit to the standard peripheral VA ECMO circuit, we were able to ensure that a sufficient amount of oxygenated blood was reaching the aortic arch as well as perfusing distally. Another advantage of VAV ECMO is the ability to achieve higher flow rates (over 5 LPM) through the VAV ECMO circuit to better oxygenate the patient with high cardiac output as was the case in our patient. Our patient had sustained cardiac output of 7-10 LPM which led to hypoxemia after the VA circuit was removed and ECMO flows were limited to 4.6 LPM through the VV circuit.

As our patient's cardiomyopathy improved well before his ARDS, the two circuits were able to be discontinued in a stepwise fashion. VA ECMO decannulation was performed on ECMO day 2, and he was transitioned completely to the VV ECMO circuit, which remained in place for an additional 9 days to support him through his severe ARDS. By placing both circuits simultaneously upon the patient's admission, we were able to minimize the number of aerosolizing procedures and thus the exposure of physicians and staff, an important consideration while caring for COVID-19 patients.

Our patient successfully recovered, and despite the severe ARDS with stress cardiomyopathy requiring ECMO therapy, our patient was able to discharge home from the ICU on hospital day 18. The relative benefit of broad-spectrum antimicrobial therapy, hydroxychloroquine, IL-6 inhibition, and ECMO support to allow the lung to recover at rest ventilator settings is unclear. More research is needed to clarify the role of medical and invasive extracorporeal oxygenation support in severe ARDS and particularly in COVID-19 ARDS.

## 4. Conclusion

As the current global SARS-CoV-2 pandemic unfolds, the natural history of the disease process is gradually being clarified. In our patient, who presented with concomitant severe ARDS and cardiomyopathy, we utilized VAV ECMO to provide both cardiac and respiratory support as he recovered from SARS-CoV-2. Although not universally accessible in large tertiary and quaternary centers, this appears to be a viable option that should be considered for COVID-19 patients with similar presentations.

## Abbreviations

ECMO: Extracorporeal membrane oxygenation
VV: Venovenous
VA: Venoarterial
VAV: Venoarteriovenous
ARDS: Acute respiratory distress syndrome.
